# Battery-Free Wireless Light-Sensing Tag Based on a Long-Range Dual-Port Dual-Polarized RFID Platform

**DOI:** 10.3390/s22134782

**Published:** 2022-06-24

**Authors:** Mahmoud Wagih, Alex S. Weddell, Steve Beeby

**Affiliations:** 1 School of Electronics and Computer Science, University of Southampton, Southampton SO17 1BJ, UK; asw@ecs.soton.ac.uk (A.S.W.); spb@ecs.soton.ac.uk (S.B.); 2 James Watt School of Engineering, University of Glasgow, Glasgow G12 8QQ, UK

**Keywords:** antennas, differential RFID, impedance matching, Internet of Things, light sensing, microstrip antennas, multi-port RFID, patch antennas, RFID, wireless sensor node

## Abstract

Radio frequency identification (RFID) represents an emerging platform for passive RF-powered wireless sensing. Differential Multi-port RFID systems are widely used to enable multiple independent measurands to be gathered, or to overcome channel variations. This paper presents a dual-port/dual-integrated circuit (IC) RFID sensing tag based on a shared aperture dual-polarized microstrip antenna. The tag can be loaded with different sensors where the received signal strength indicator (RSSI) of one IC is modulated using a sensor, and the other acts as a measurand-insensitive reference, for differential sensing. The 868 MHz tag maintains a minimum unloaded read range of 14 m insensitive to deployment on metals or lossy objects, which represents the longest reported range of a multi-port RFID sensing tag. The tag is loaded with a light-dependent resistor (LDR) to demonstrate its functionality as a battery-less wireless RFID light sensor. Following detailed RF characterization of the LDR, it is shown that the impedance, and consequently the RSSI, of the sensing tag are modulated by changing the light intensity, whereas the reference port maintains a mostly unchanged response for a correlated channel. The proposed tag shows the potential for channel variations-tolerant differential RFID sensing platforms based on polarization-diversity antennas.

## 1. Introduction

With wireless sensors finding applications in industrial [1], healthcare [2], and environmental monitoring [3], a plethora of radio frequency identification (RFID)-based sensing systems have been developed. The ultimate goal of low-cost, low-complexity wireless sensors is to maintain a robust sensing performance, without the need for batteries, over a long read range [4].

RFID integrated circuits (ICs) utilize RF wireless power transmission (WPT) as a key enabler, where integrated on-chip rectifiers, designed for high-sensitivity, provide µW-level power to the back-scattering transponder [5]. Subsequently, RFID antennas are in essence a class of complex-conjugate rectennas [6], designed to directly match the on-chip rectifier and modulator. In contrast to chipless RFID [4,7], RFID interrogation is globally regulated, in terms of frequency bands and radiated power, and is commercialized in retail applications, lowering the barrier to wide-scale adoption of RFID wireless sensing [8]. Moreover, many RFID tags have been developed for a variety of implementations including bio-compatible [9], wearable [10], and washable [11] materials, enabling RFID sensing to be integrated in different applications.

A variety of Ultra-High Frequency (UHF) Rain^TM^ [12], as well as chipless [4], RFID sensors have been realized based on integrating a sensing material that responds to external stimuli such as gases, humidity, or pH within the antenna. The sensor’s response can then be read through the tag’s received signal strength indicator (RSSI) [13], or in the case of chipless RFID, through the broadband transmission response influenced by the radar cross-section (RCS) [7]; however, using the magnitude of the RSSI introduces channel-dependent interference. Some sensors overcome the magnitude’s fluctuations by interrogating the resonant frequency using a broadband threshold power or RSSI measurement [14]. Nevertheless, this approach requires a broadband reader that may not be permitted by local spectrum regulations, which limits the interrogation to either the 868 or 915 MHz band, with a bandwidth under 5 MHz; therefore, RSSI-based sensing is the most practical from a regulations point of view [13], as well as for compatibility with existing low-cost RFID readers. In order to overcome the distance-induced variations in the RSSI, a reference tag, not loaded with any sensing elements, is typically required [15].

Differential and multi-port RFID systems were proposed as a solution to robust RFID sensing [16]. Compared to multiple co-located tags, each having an individual antenna [17], realizing a multi-port single-antenna RFID tag with several RFID integrated circuits (ICs) improves the integration as well as results in a similar far-field response. Differential RFID sensing platforms have also been developed for epidermal devices, tuned for operation in direct contact with the skin, enabling two measurands to be detected simultaneously [18]. Nevertheless, the majority of existing RFID sensing tags are still based on “wire-type” dipole antennas [3,16], which results in a reduced read range when operating near a lossy medium, due to the high sensitivity of their input impedance to the surroundings. Furthermore, the unloaded read range of multi-port RFID tags is often lower than their conventional counterparts.

In this paper, we propose a long-range RFID sensing platform based on a dual-IC microstrip patch antenna, achieving a best-in-class unloaded read range exceeding 14 m. The tag enables different resistive or capacitive sensors to be added as a loading element to one IC’s feed, to act as a sensor, while the other IC remains unloaded acting as a reference, minimizing the high distance and channel sensitivity of RFID sensors. Demonstrated as a battery-less wireless light sensor based on a commercial light dependent resistor (LDR), the proposed tag shows that future RFID sensors can be realized based on polarization-diversity multi-port antennas.

## 2. Differential RFID Sensing Tags

**Figure 1:**
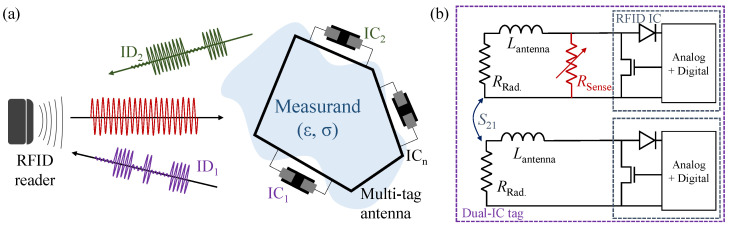
Multi-port RFID sensing systems: (**a**) a single-antenna tag with multiple unique RFID ICs and back-scattered responses [16]; (**b**) circuit model of a two-tag dual-port system with a resistive sensing element.

Figure 1a shows a typical multi-tag RFID system, where a cluster of RFID ICs interacts differently with their surroundings [16]. In a multi-tag/multi-ID system, the relative permittivity ($\epsilon_{r}$) and conductivity (σ) of the medium to be measured interacts differently with the different ports of the antenna; it was previously found that having multiple ICs can have a minimal influence on individual tags’ read range [18]. For example, a specific measurand such as humidity could influence the permittivity of a substrate, such as liquid crystal polymer, which shifts the resonant frequency of a tag [19].

As for measurands that do not directly influence the medium’s properties, the antenna can be loaded with a capacitive or a resistive sensing element, as in Figure 1b. An example of a measurand that does not introduce variations in the antenna’s electric properties is light intensity variation; therefore, a sensor, such as a commercially available discrete light-dependent resistor (LDR), can be used as a resistive sensor to modulate the light intensity variations onto the antenna’s gain, consequently varying the RSSI; however, as LDRs are typically used at DC or very low frequencies, the RF response needs to be characterized prior to integration in a sensing antenna. The LDR RF characterization is presented in Section 4.1.

**Figure 2:**
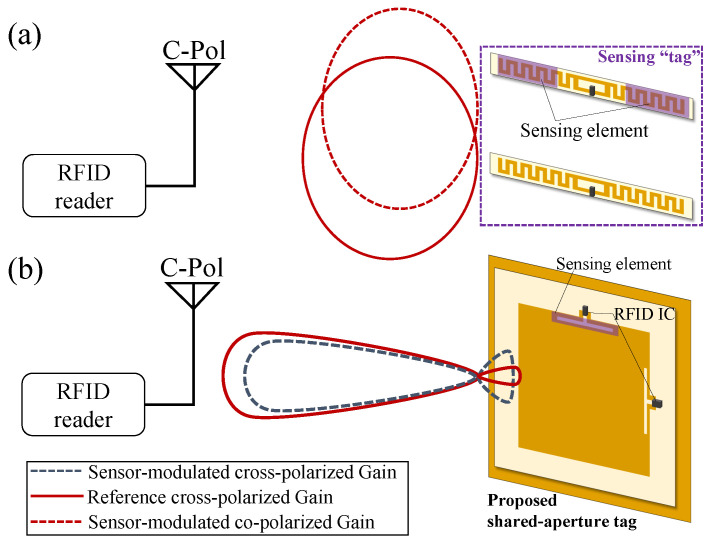
The architecture of a dual-IC sensing tag: (**a**) a conventional two-antenna design [17]; (**b**) the proposed RFID sensing tag based on orthogonally polarized “sensing” and “reference” signals using a shared-aperture antenna.

This dual-tag/dual-IC approach however relies on the antenna’s radiation patterns being stable. To explain, when interrogating the tags cluster from different angles, the changes in the differential RSSI between the RSSI will be influenced by the angular patterns of the antenna. To illustrate, Figure 2 shows the qualitative radiation patterns of two differential RFID sensing tags. In Figure 2a it can be seen that for two co-located antennas, the radiation patterns may not fully overlap, which will introduce additional uncertainty when performing differential RSSI measurements.

To overcome this problem, a shared aperture multi-port antenna, where both ports maintain a similar radiation pattern in the direction of interrogation, can be used. As with multi-antenna communication systems, e.g., multi-input multi-output (MIMO) networks, the antenna ports need to maintain high isolation (low $S_{21}$). The sensing principle of the proposed polarization diversity aperture-type RFID antenna is shown in Figure 2b. Each of the antenna’s ports is connected to a separate RFID IC, where the RSSI of both tags is expected to exhibit a similar response to distance and other obstructions in the channel, due to the similarity in the radiation patterns of the shared-aperture microstrip antenna. The sensing IC’s port, i.e., the inductive matching loop, is then loaded with a sensing material or component to modulate its impedance, while not affecting the impedance of the reference port. This will, in turn, vary the RSSI of the sensing IC but not the reference IC. Moreover, the orthogonal linear polarizations can be read using a standard circularly polarized reader.

## 3. Long-Range RFID Sensing Antenna Design

To realize a scalable and long read range differential RFID platform, the key antenna design requirements for the proposed sensor are:

1. A differential complex *Z* realized using a feed with scalable geometry, to match the complex *Z* of different off-the-shelf RFID ICs or rectifiers;
2. Similar gain patterns with orthogonal and high-purity polarization across both ports, to reduce the envelope correlation coefficient (ECC);
3. High port-isolation to ensure the reference tag remains unaffected by the measurand modulating the sensor’s impedance and RSSI.

To achieve a higher gain than conventional RFID tags, the proposed dual-port tag is based on an inductive-fed microstrip patch antenna [20]. Moreover, using a microstrip antenna-based tag enables the sensor to be placed on metals as well as near lossy objects such as fluids or the human body. With two orthogonally polarized ports, the two ICs are expected to maintain high isolation (low $|S_{21}|$), as well as stable radiation patterns. The microstrip patch is fed using a scalable loop to realize an inductive input impedance, which can be tuned to match the RFID IC’s capacitive $Z_{\mathrm{in}}$ [20].

**Figure 3:**
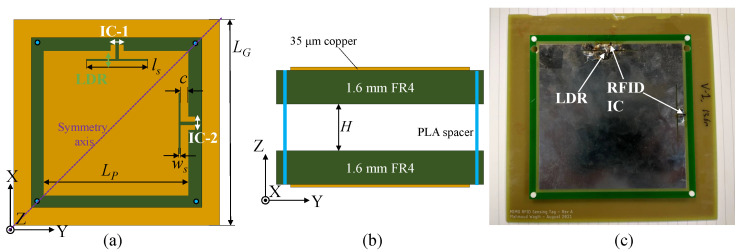
The proposed dual-IC long-range tag: (**a**) XY layout and dimensions (in mm); (**b**) XZ layout; (**c**) photograph; $L_{P}$ = 115, $L_{G}$ = 160, $l_{s}$ = 46, $w_{s}$ = 1.0, *c* = 5.0, *H* = 10.

Figure 3 shows the layout and dimensions of the proposed antenna, as well as a photograph of the fabricated prototype. The prototype is realized using an inexpensive FR4 substrate ($\epsilon_{r}\approx$ 4.2, tanδ≈ 0.02), where the radiating patch is separated from the ground plane by a 3D-printed spacer. Using a variable-height spacer, the impedance of the antenna can be fine-tuned post-fabrication to mitigate variations in the PCB manufacturing process, which arises from uncertainty around the relative permittivity of low-cost commercial FR4 boards. The RFID IC used in this work is the NXP UCODE 7, owing to its high read sensitivity with a minimum activation power of −21 dBm. Nevertheless, as the antenna’s input impedance can be varied by changing the slots’ dimensions [20], other RFID ICs can be matched using the same antenna design, including self-tuning RFID ICs, which enable the read range to be maintained.

## 4. Sensor Tag Characterization

### 4.1. LDR RF Characterization

**Figure 4:**
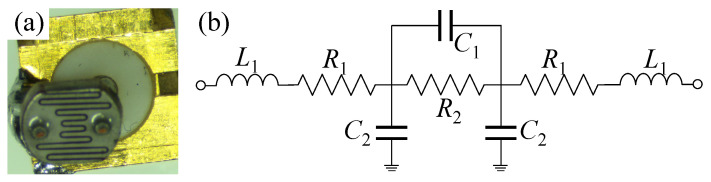
The RF-readout LDR: (**a**) the LDR-under-test mounted on an RF connector; (**b**) equivalent circuit model of the LDR.

The selected LDR is the NSL-6112 from Advanced Photonix, chosen for having a very low minimum resistance, which will resemble a response close to a short circuit when the light intensity increases. As the LDR comes in a through-hole package, unsuitable for RF applications, the leads have been trimmed off to minimize the series inductance and enable the change in the resistivity to have a more observable effect in the RFID frequency band. For initial characterization, the LDR was mounted on an RF SMA connector to measure its RF input impedance, as shown in Figure 4a.

**Figure 5:**
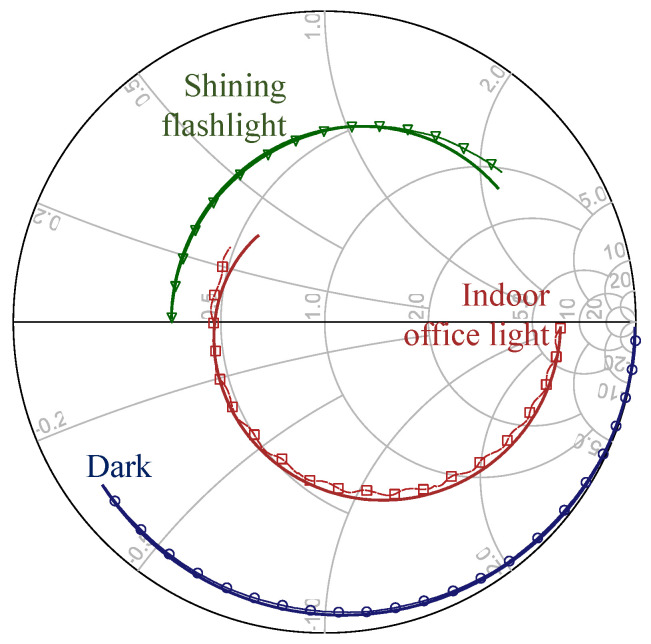
Measured (solid with markers) and calculated (solid) impedance response, from 150 kHz to 1.2 GHz, of the LDR and its extracted equivalent circuit model under different illuminations.

The DC to RF response of the SMA-mounted LDR was measured using a calibrated Rohde and Schwarz ZVB4 Vector Network Analyzer (VNA) from 150 kHz to 1.2 GHz. First, the input power level was swept from −10 up to 15 dBm to verify the linearity of the LDR, where the input impedance was found to be maintained regardless of the input power level. The input impedance ($Z_{11}$) of the LDR was measured at three different illuminations: (a) in the dark (covered by a black low-dielectric foam), (b) in indoor natural lighting, and (c) under a bright flashlight. The light intensity in each scenario was measured using a CEM DT-1309 Lux meter, and is given in Table 1. As the lux meter and the chosen LDR have varying spectral ranges, and the light intensities investigated include different light sources, the indicated values represent an estimate of the light intensity available, as opposed to the exact intensity at which the sensor is calibrated. Figure 5 shows the Smith chart plot of the measured impedance response, exhibiting significant changes at all frequencies in response to the varying light intensities.

To enable the LDR to be modeled and incorporated in different antenna models, an equivalent circuit model was extracted to fit the measured impedance response. Figure 4b shows the two-port “lumped” device model, widely used to model individual components [21]. The values of the equivalent circuit model components are summarized in Table 1. As observed in Figure 5 the calculated and measured $Z_{11}$ response of the LDR is in very good agreement, which enables the LDR model to be used to predict its performance. Moreover, it shows that the simple two-port device model [21], in Figure 4b, is adequate for modeling resistive sensors for sub-1 GHz RF sensing applications. The modeled and measured series DC resistive element, $R_{2}$, is in good agreement with the resistance variation range indicated in the manufacturer’s specification.

### 4.2. RFID Antenna Simulation and Measurements

**Figure 6:**
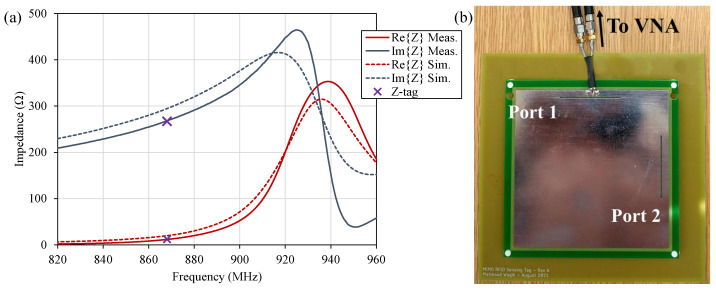
(**a**) Simulated (dashed) and measured (solid) complex impedance of the antenna matching the tag around 868 MHz; (**b**) photograph of the tag’s measurement setup using a balanced coaxial jig.

The input impedance of the proposed antenna, before loading with any sensing elements, has been simulated in CST Microwave Studio (Finite Difference Time-Domain, FDTD) and measured experimentally using a two-port VNA with a differential coaxial jig with a common ground. This approach is widely used to measure the complex input impedance of RFID antennas [11] as it enables broadband balun-free measurement of balanced loads [22]. The VNA was calibrated using the standard two-port Through, Open, Short, and Match (TOSM) calibration, followed by an automated port extension to de-embed the phased delay and insertion losses in the coaxial jig. Figure 6a shows the simulated and measured input impedance of the antenna, designed to present a complex-conjugate match to the IC, with a photograph of the measured prototype with the balanced jig shown in Figure 6b. As observed in the impedance plot, the antenna maintains a very close match to the complex-conjugate of the IC’s datasheet impedance, which implies minimal reflection between the IC and the antenna leading to a long read range. The discrepancy observed between the simulated and measured responses could be attributed to variations in the FR4 $\epsilon_{r}$ as well as tolerances in the assembly of the tag with the 3D-printed spacer between the patch and its ground plane.

In addition to simulating the impedance response of the antenna, in good agreement with the measured impedance, the far-field properties were simulated at 868 MHz. The antenna’s peak simulated gain was found to be 5.3 dBi, which includes the dielectric and conductive losses. The read range of the tag has been characterized indoors, using a commercial RFID reader (handheld Zebra RFD8500), at 868 MHz (the EU RFID band) with an equivalent isotropic radiated power (EIRP) of 33 dBm. As the reader uses a circularly polarized antenna, the co-linearly polarized component will be 50% of the circularly polarized EIRP, i.e., 30 dBm. The theoretical free-space read range *r* can be calculated using

(1) $$r=\frac{\lambda }{4\pi }\sqrt{\frac{G_{r}G_{t}P_{t}}{P_{th}}},$$

assuming no impedance mismatch between the IC and the antenna. $G_{r}$ is the antenna’s simulated gain (5.3 dBi), $G_{t}P_{t}$ are the 30 dBm (1 W) co-polarized EIRP, and $P_{th}$ is the IC’s sensitivity (−21 dBm). Table 2 shows the calculated and measured read range of the tag. Despite the antenna being symmetric, and having the same simulated and measured input impedance at both ports it was found that one IC was readable up to 18 m away from the reader. The variation in the read range could be attributed to tolerances in the manual assembly of the tag, where the RFID IC’s input impedance can be influenced by the capacitance (<1 pF) between the soldering pads and the unconnected additional contacts on the IC’s package.

**Figure 7:**
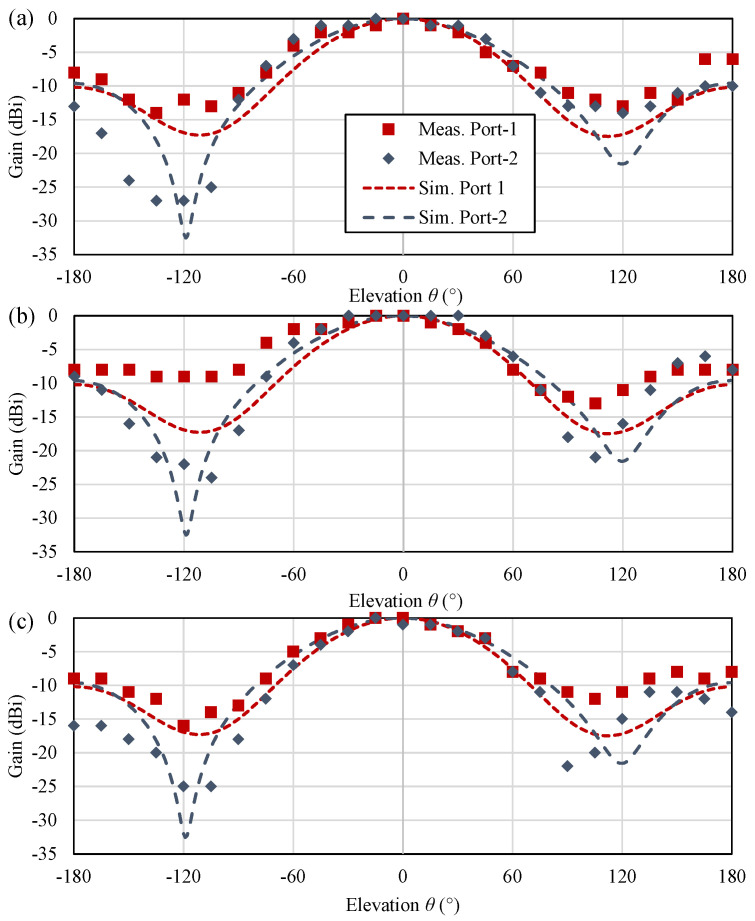
Simulated and measured (using the RSSI) radiation patterns of the proposed RFID sensing platform: (**a**) in space; (**b**) on a lossy fluid (ethanol); (**c**) mounted on a metal surface.

To achieve a stable sensory response across both ports, similar gain patterns need to be maintained in the direction of interrogation. The “digital” radiation patterns of the antenna were measured using the RSSI reported by the RFID reader across both ports, while the antenna was rotated around its elevation (YZ) plane in an indoor environment. The antenna was positioned 1 m away from the reader to ensure operation in the far-field region. Utilizing the RSSI to reconstruct the radiation patterns in a realistic operation environment represents a more realistic test, which includes the effects of the IC mounting, matching, as well as the quantization effect introduced by the RSSI error in commercial readers. Figure 7 shows the RSSI pattern of both ICs, as well as the CST-simulated radiation pattern of the antenna on both ports.

The measured radiation patterns in Figure 7a exhibit a good agreement with the simulation, even in the antenna’s back lobe, which is expected to be filled by multi-path reflections in the echoic measurement environment. Moreover, both ICs exhibit a similar gain pattern in the antenna’s −3 dB broadside beamwidth; therefore, the tag’s differential RSSI will be consistent for elevation angles between −30${}^{\circ }$ and 30${}^{\circ }$. To validate the tag’s insensitivity to mounting on lossy objects, the patterns have been remeasured over a lossy dielectric, i.e., a plastic container filled with ethanol. With a tanδ> 0.4 around 868 MHz, this test represents a conservative estimate for the antenna’s performance on lossy objects including fluids [14], the human body [20], as well as food products [23]. Figure 7b shows the measured patterns of the tag over the lossy dielectric, showing no variations in the main lobe. This is due to the shielding offered by the ground plane of the tag; neither IC observes any noticeable difference in the main lobe.

As the antenna is backed by a ground plane, it can be used on metal objects with no influence on its radiation patterns. Figure 7c shows the measured patterns of the antenna over a 30 × 21 cm metal sheet, where it can be seen that the antenna’s main lobe very closely matches the simulated response. Moreover, the side lobe of the antenna is suppressed as most of the power diffracting from the ground plane’s edges is now reflected to the broadside direction; therefore, the proposed tag is suitable for mounting on different objects, with no influence on its sensory response and far-field properties.

**Figure 8:**
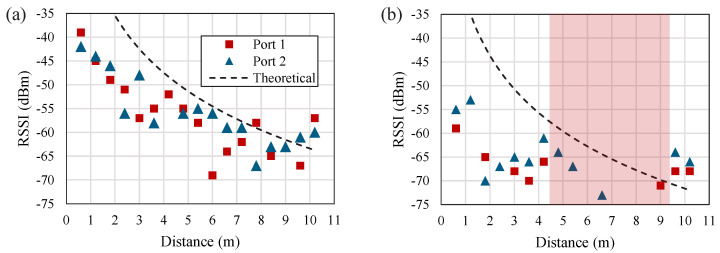
Measured RSSI over distance of the two RFID ICs integrated in: (**a**) the proposed high-gain patch antenna, (**b**) two co-located dipoles based on [11] separated by 5 cm; the shaded region in (**b**) indicates the “blind-spot” where at least one IC is not readable.

Following the characterization of the radiation patterns, the RSSI variation over distance were investigated. For benchmarking, two co-located dipole-based tags, based on [11], were used and co-located with a 5 cm antenna clearance. Figure 8 shows the measured RSSI over distance for both the dual-polarized ICs, and the reference dipoles. Comparing the response of the low-gain dipoles and the proposed tag, the benefits of the high-gain design manifests in an improved reading reliability. To explain, at least one of the dipole-based tags becomes unreadable for distances between 4.5 and 9 m, which can be attributed to destructive interference from multi-path reflections.

While the variation in the measured RSSI across both ports of the proposed antenna can be attributed to variations in the horizontally and vertically polarized channels, a comparable variation is observed for the co-polarized dipole tags; therefore, the variation in the differential RSSI can be regarded as the minimum sensor dynamic range required to resolve the tag’s sensory response. For example, for distances under 2 m, it can be seen that the proposed tag’s ports maintain a similar RSSI with under 2 dB discrepancy in the differential response; therefore, a sensor with a >2 dB dynamic range could be used in these operation conditions.

### 4.3. Light-Sensing Differential RFID Tag

To realize the light-sensing tag, the sensing port has been loaded with the commercial LDR (Advanced Photonix NSL-6112) soldered across the center of the inductive matching loop. As the LDR’s $Z_{11}$ (in Figure 5) varies in response to light, the antenna’s *Z* at the LDR-loaded port will change. On the other hand, the reference port should maintain its original impedance regardless of the variations in the light intensity, owing to the high isolation between both ports.

**Figure 9:**
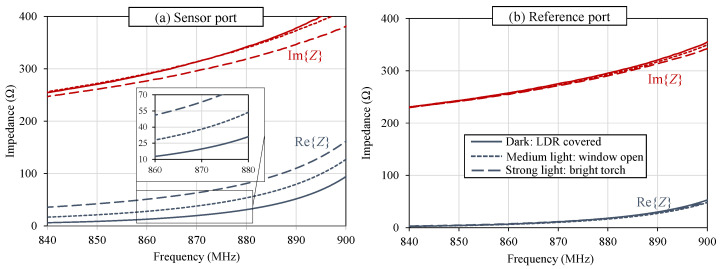
Measured impedance of the LDR-loaded light-sensing antenna: (**a**) measurand-modulated port; (**b**) unchanged reference port.

The sensing tag was characterized in the same three conditions: in the dark, in indoor natural lighting, and under a flashlight. $Z_{\mathrm{antenna}}$ was measured across both ports under the three illumination conditions, and is shown in Figure 9a,b, for the sensing and reference ports, respectively. In Figure 9a, the antenna’s ℜ{Z} and ℑ{Z} vary in response to the changing light intensity, with the largest variation observed in ℜ{Z}. Recalling Table 1, the largest change in the LDR’s impedance response is observed in $R_{2}$, which is a real impedance term, as opposed to the capacitance terms, which observe smaller variations. On the other hand, the reference port, in Figure 9b, shows an unaffected response, demonstrating that its impedance matching, radiation, and subsequently RSSI will not vary for different light conditions.

To validate the real-world performance of the sensor, the dual-IC tag has been loaded with the LDR in the same position and interrogated using the RFD8500 reader. Five RSSI readings were obtained and averaged to overcome the 1 dB quantization in the RSSI values quoted by the reader. Moreover, it is important to note that for the handheld reader, and for most low-cost receivers, the RSSI is given with at least ±1 dB accuracy, which is expected to further influence the measured response. The RSSI was measured at three read ranges for dark and light conditions, corresponding to the LDR impedances for dark and office light in Table 1, respectively. Although the LDR’s RF response under direct illumination from a flashlight, a similar measurement setup was not possible with the RFID antenna, as the flashlight will interfere with the antenna’s near- and far-fields. The wireless measurement range was limited to the maximum range at which both ICs were readable, in either light or dark conditions.

The measured RFID RSSI results are shown in Table 3. Across all distances, the RSSI of the sensing IC decreases by 1–3.4 dB, whereas the reference IC’s RSSI remains mostly unchanged. This validates the tag’s ability to differentiate between the reference and sensing ports/ICs, as previously observed in the measured impedance response in Figure 9; therefore, it is demonstrated that the proposed shared-aperture tag could be used simultaneously as a sensor and a reference in RFID sensing.

### 4.4. Discussion and Comparison

In Table 3, it was seen that the proposed antenna can be used for wireless light-sensing over a relatively short read range. For a longer sensor-loaded read range to be achieved, the antenna will need to be designed for the specific sensor used. For example, this can be achieved using the LDR’s equivalent circuit model from Figure 4. Nevertheless, the proposed tag, despite the introduced mismatch at the LDR-loaded port, maintains a state-of-the-art loaded read range, as seen in Table 4.

From the table, it can be seen that the antenna maintains the longest unloaded read range of a multi-port multi-IC tag, showing the potential for improving the read range of differential RFID-enabled sensing tags by over 2× compared to state-of-the-art sensing antennas. The read range of the sensor-loaded port was limited to approximately 2 m, as the antenna design did not consider the introduced impedance change by the LDR; however, the sensing read range of the antenna is comparable to previously reported sensors, as in Table 4. Moreover, as observed in Figure 8, the proposed antenna maintains an improved reading reliability, compared to a long-range dipole [11], where a “blind-spot” of over 4 m resulted in either one or both of the ICs being unreadable. This demonstrates that the use of low-gain dipole antennas for differential sensing is not suitable for long operation ranges.

In the proposed tag, a resistive element was used as a sensor, for the first time, showing that varying DC resistance of a discrete component can translate to significant changes in the RF response, as previously observed in Section 4.1; however, as the tag was interrogated at a single frequency and using a low-resolution reader, the RSSI exhibited limited variations, as in Table 3; therefore, the interrogation of the proposed RFID sensing tag can be improved by using a higher-accuracy wide-band reader, which can interrogate the tags across the 860–940 MHz spectrum [18]. To explain, by measuring the turn-on power threshold of the tags [18], both the gain and the resonant frequency could be interrogated. Moreover, by measuring the turn-on power threshold of the tags, the multi-path effects that might affect the backscattered RSSI accuracy can be avoided.

As a result, the other sensors compared in the table such as humidity-sensitive polymers (PEDOT:PSS) [25] and thermal insulators [18] can be used to realize sensors aimed at other applications using the proposed antenna. Finally, while a rigid PCB was used to demonstrate this proof-of-concept, a range of fabrication techniques including screen printing [21] or photolithography on ultra-thin flexible polyimide laminates [11,13] can be used to realize the proposed design on inexpensive flexible or bio-degradable substrates.

## 5. Conclusions

In this paper, a dual-port RFID sensing platform was proposed for the first time based on a shared-aperture microstrip patch antenna. By loading the sensing IC’s feed with a sensing component or material, and leaving the reference port unloaded, differential RFID sensing can be used to achieve robust sensing as well as enable multi-measurand detection. The tag maintains an unloaded read range over 14 m, owing to the high antenna gain over 5 dBi, a longer range compared to state-of-the-art multi-IC RFID sensors. The proposed tag is demonstrated in a light-sensing application based on a commercial LDR, where it is possible to modulate the impedance and subsequently the RSSI of the sensing port but not the reference port. Following a detailed characterization of the commercial LDR, it was found that off-the-shelf resistive sensors, not designed or packaged for RF applications, could still be used in RF sensing applications including battery-less RFID tags. It is anticipated that the proposed RFID sensing antenna will enable a plethora of channel/distance-resilient sensing applications based on existing smart sensing materials.

## Figures and Tables

**Table 1:** The LDR’s DC to UHF equivalent circuit model under different illuminations.

	$L_{1}$ (nH)	$R_{1}$ (Ω)	$R_{2}$ (Ω)	$C_{1}$ (pF)	$C_{2}$ (pF)	Meas. $Z_{\mathrm{in}}$ *	Calc. $Z_{\mathrm{in}}$ ${}^{†}$
Dark (<1 Lux)	2.0	2.0	40k	2.1	1.3	3.55 − j59.8	3.08 − j38
Office light (≈614 Lux)	3.8	9	345	2.9	1.3	24.9 − j20.5	23.4 − j10.9
Flashlight (>13,000 Lux)	4.6	4	9	2.9	1.3	28.7 + j64.4	27 + j58.8

* Measured $Z_{\mathrm{in}}$ (Ω) at 868 MHz; ^†^ equivalent circuit $Z_{\mathrm{in}}$ (Ω) at 868 MHz.

**Table 2:** Calculated and measured indoor read range of the proposed flexible RFID tag.

Frequency (MHz)	Calculated Range	Measured Range
868	17.4 m	18 m (IC 1); 14 m (IC 2)
915	18.0 m	-

**Table 3:** Performance of the RFID-enabled distance-tolerant light sensor.

Condition ${}^{*}$	Distance	Sensor RSSI	Ref. RSSI	Diff. RSSI
Dark (<1 Lux)	0.5 m	−38 dB	−41 dB	3 dB
Light (≈600 Lux)	0.5 m	−39.8 dB	−41 dB	1.2 dB
Dark (<1 Lux)	1.1 m	−41 dB	−42 dB	1 dB
Light (≈600 Lux)	1.1 m	−42 dB	−42 dB	0 dB
Dark (<1 Lux)	1.5 m	−49.4 dB	−43.5 dB	−5.9 dB
Light (≈600 Lux)	1.5 m	−52.8 dB	−44 dB	−8.8 dB

* Dark: corresponds to the LDR covered with a black low-permittivity foam; Light: corresponds to the office light condition.

**Table 4:** Comparison of the proposed RFID sensing platform with state-of-the-art differential and multi-port RFID sensors.

	Antenna Design	Unloaded Read Range	Application	Sensor	Materials	Dimensions (cm)
This work	Dual-pol. inductive microstrip patch	14–18 m (1.5 m sensing range)	Light-sensing	Resistive: LDR	FR4 PCB with printed spacer	16 × 16 × 1
2022 [17]	Dual RFID dipoles	3 m (0.5 m sensing range)	Human activity tracking	Mechanical: stretchable antenna	Embroidered antenna on textile	9 × 3 (individual) >9 × 7 (combined ${}^{*}$)
2021 [18]	Dual-pol. wire antenna	0.6 m (measured); estimated 1.5 m using a more sensitive IC	Temperature sensing	Dielectric: thermal insulation	Flexible copper on silicone	3.5 × 3.5
2012 [3]	Dual dipole antennas	8 m (3.5 m calculated sensing range)	Humidity sensing	Lossy dielectric (resistive): PEDOT:PSS	Copper on Teflon	4.8 × 5.8 × 0.4
2011 [24]	Dual-IC dipole antenna	1.5 m	Thermal threshold sensing	Mechanical: shape- memory alloy	FR4 PCB	8 × 5.8 × 0.6

* Estimated from the photograph of the two-antenna sensing module.

## Data Availability

Datasets supporting this paper will be made available from the University of Southampton repository at DOI: https://doi.org/10.5258/SOTON/D2275.
